# Gold nanobipyramids doped with Au/Pd alloyed nanoclusters for high efficiency ethanol electrooxidation†

**DOI:** 10.1039/d1na00878a

**Published:** 2022-03-03

**Authors:** Baihe Hanqi, Juan Xu, Xingzhong Zhu, Caixia Kan

**Affiliations:** College of Science, Nanjing University of Aeronautics and Astronautics Nanjing 211106 China cxkan@nuaa.edu.cn; MIIT Key Laboratory of Aerospace Information Materials and Physics, Nanjing University of Aeronautics and Astronautics Nanjing 211106 China

## Abstract

Plasmonic metal nanostructures are of great interest due to their excellent physicochemical properties and promising applications in a wide range of technical fields. Among metal nanostructures, bimetallic nanostructures with desired morphologies, such as core–shell, uniform alloy and surface decoration, are of great interest due to their improved properties and superior synergetic effects. In this paper, Au/Pd nanoclusters were deposited on the surface of gold nanobipyramids (AuBPs) into a core–shell nanostructure (AuBP@Au_*x*_Pd_1−*x*_) through a reductive co-precipitation method. The AuBP@Au_*x*_Pd_1−*x*_ nanostructure integrates effectively the advantages of plasmonic AuBPs and catalytic Pd ultrafine nanoclusters, as well as the stable Au/Pd alloy shell. The AuBP@Au_*x*_Pd_1−*x*_ nanostructure exhibits superior electrocatalytic activity and durability for oxygen reduction in alkaline media owing to the synergistic effect between the AuBP core and Au/Pd shell. Furthermore, the shell thickness of AuBP@Au_*x*_Pd_1−*x*_ nanostructures can be adjusted by varying the amount of precursor. Overall, the catalytic activity of bimetallic Au/Pd catalysts is likely to be governed by a complex interplay of contributions from the particle size and shape.

## Abstract 2

AuBPs@Au_*x*_Pd_1−*x*_ nanocatalysts were synthesized as a promising electrocatalyst for ethanol fuel cells.

## Graphical abstract

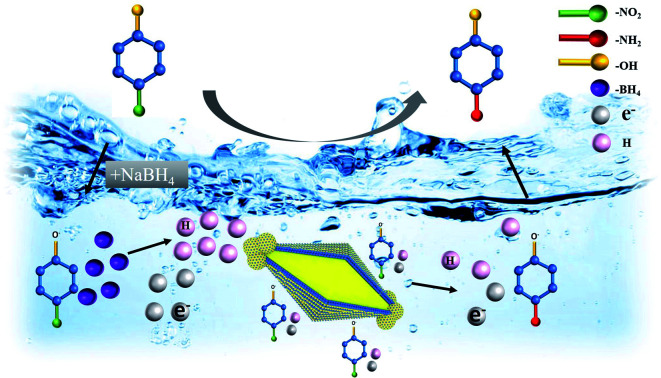

## Introduction

The use of small molecule alcohols (*e.g.* ethanol) as alternatives to current hydrocarbon fuels has attracted considerable attention.^1,2^ Because ethanol is an environmentally friendly energy conversion agent with fast kinetic reactions and low corrosiveness, it can be used in alkaline media. In addition, it is extremely suitable for use with other catalysts, especially non-Pt catalysts.^3,4^ Compared with Pt-based catalysts, Pd is more active and stable and its price is half the current market price of Pt; it is widely available and has the potential to replace Pt for various energy-related reactions in alkaline media. In most cases, optimization of Pd-based alloys (such as alloying Pd with Ni, Co, Cu, Au, and Ag) and construction of novel metallic nanostructures have greatly enhanced their specific physical and chemical properties.^5–8^ It is reported that Pd electronic characteristics can be tailored by the alloyed metals and nanostructure morphologies.^9^ Pd electron regulation can not only optimize its binding energies to reactive species and intermediates, but can also improve its catalytic activity and stability. Thus adjusting the electronic properties of Pd catalysts by constructing bimetallic alloy nanostructures provides an effective method to improve the catalytic performances.^10–14^

In general, bimetallic nanocatalysts mainly include plasmonic and catalytic metals. The former one (such as Au, Ag and Cu) represents excellent surface plasmon related effects and the latter provides superb enhancement in activity and selectivity.^15–17^ Particularly, plasmonic nanostructures with elongated morphologies, such as Au nanorods (AuNRs) and Au nanobipyramids (AuBPs), are of great interest because of their tunable surface plasmon resonance (SPR) properties.^18^ Due to their sharp ending structure, AuBPs have a strong ability in exciting SPR with a high quality factor and enhanced local electron field, which is advantageous for the catalysis reaction process. To improve catalyst properties, another key issue is enlarging the specific surface areas. Therefore, catalytic metal nanoframes and monoatoms have attracted attention because of their high specific surface areas.^19–22^ The stability of open nanoframe structures formed by the sacrificing Ag template method needs to be improved. The catalyst deactivation of monoatomic catalysts and the selection of specific supports are also particularly challenging. Consequently, effectively managing the stability and surface area of catalysts is also worth studying. This is especially true for supported catalysts, in which the morphology of the catalyst will greatly affect the catalytic activity and their use as efficient electrocatalysts.

In recent years, Pd-based nanostructures have drawn considerable attention in electrocatalyst utilization.^23^ It is well known that different preparation methods produce nanostructures with different catalytic activities.^24^ Consequently, optimal catalytic performance is often achieved by careful selection of the preparation method. For example, Yang's group reported that Au@Pd core–shell nanobricks with a concave surface exhibited significantly higher electrocatalytic activity for the ethanol oxidation reaction than the commercial black Pd.^25^ Yin *et al.* synthesized spikes-like nanostructures by combining Pd nanohumps with Au nanowires through a simple wet chemical method.^26^ In Zhang's study, enhanced electrocatalytic activity was ensured for the series of Pd_*x*_Ag_*y*_ nanoparticles by the coreduction strategy.^27^ Park and Im's group demonstrated the synthesis of flower-like Au@Pd core–shell nanostructures by using a facile chemical method, and the structures improved electrocatalytic activity for the ethanol oxidation reaction in an alkaline solution.^28^ Besides, the large specific surface area of monoatomic catalysts improves their activity, but they are limited by the poor stability of the carrier. And the catalyst durability restricts further application.^29^ In the designing and developing catalysts with high efficiency for the ethanol electrooxidation reaction in ethanol fuel cells, the application of Au based nanostructures has been plagued by low catalytic activity, poor usage and high price. In addition, the efficient utilization of Pd in the above catalysts is critical for their practical application. Therefore, the design and synthesis of new type Pd-based catalysts for further improving their catalytic performances is still an attractive research topic.

In our recent study of plasmonic AuBPs, it is found that catalytic metal cluster arrays and bimetallic nanoframes can be precisely controlled on the surface of AuBPs.^30,31^ Herein, Au/Pd alloy clusters with different concentrations have been deposited on the surface of as-prepared AuBPs into AuBP@Au_*x*_Pd_1−*x*_ nanostructures. The obtained nanostructures showed plasmonic optical properties, high stability and promising electrocatalytic performance. With increasing the Pd content, the Pd-enriched nanoclusters are attached to the surface of highly stable AuBPs, which endows the AuBP@Au_*x*_Pd_1−*x*_ nanocatalysts with a large specific surface area. The AuBP@Au_*x*_Pd_1−*x*_ nanostructures integrate effectively the advantages of plasmonic AuBPs and catalytic Pd ultrafine nanoclusters, as well as the stable Au/Pd alloy, exhibiting superior electrocatalytic activity and durability for oxygen reduction in alkaline media. The catalytic properties of Au/Pd nanostructures were evaluated using a model reaction based on the reduction of 4-nitrophenol (4-NP) to 4-aminophenol (4-AP) by NaBH_4_. The electrocatalytic activities were measured by cyclic voltammetric measurements and durability tests were carried out in alkaline solution.

## Experimental

### Materials

Hexadecyltrimethylammonium bromide (CTAB, 99%) and cetyltrimethylammonium chloride (CTAC, 99%) were obtained from Alfa Aesar. Hydrogen tetrachloroaurate tetrahydrate (HAuCl_4_·4H_2_O, 99%), silver nitrate (AgNO_3_, 99.8%), palladium chloride (PdCl_2_, 99%), sodium borohydride (NaBH_4_, 99%), 4-nonylphenol (4-NP, 99%) and l-ascorbic acid (AA, 99.7%) were purchased from Sigma-Aldrich. Deionized water with a resistivity of 18.2 MΩ cm produced by a Direct-Q 5 ultraviolet water purification system was used in all experiments.

### Synthesis of Au nanobipyramids (AuBPs)

The seed-mediated growth method was used to prepare AuBPs as reported previously.^30,31^ In a typical procedure, this experiment is divided into two steps. Firstly, the seed solution was prepared by adding an ice-cold NaBH_4_ solution (0.01 M, 0.2 mL) into a mixed solution of HAuCl_4_ (0.01 M, 0.2 mL), trisodium citrate (0.01 M, 0.25 mL) and deionized water (9.625 mL) under vigorous stirring. The solution was placed in an oven kept at 55 °C for 2 hours. Secondly, 40 mL cetyltrimethylammonium bromide (CTAB) solution (0.1 M) was mixed with HAuCl_4_ (2 mL, 0.01 M), AgNO_3_ (0.01 M, 0.4 mL), HCl (1 M, 2 mL), ascorbic acid (0.1 M, 0.3 mL) and the as-prepared seed solution (0.6 mL) in an appropriate order. The mixed solution was shaken at 35 °C for 2 hours for the formation of AuBPs.

### Synthesis of AuBP@Au_*x*_Pd_1−*x*_ nanostructures

The formation of the AuBP@Au_*x*_Pd_1−*x*_ nanostructures was carried out by the simultaneous addition of HAuCl_4_ (0.4 mL YmM) and H_2_PdCl_4_ (0.4 mL ZmM) at 0.01 mL min^−1^ at 65 °C into a AuBP colloid which redispersed in a cetyltrimethylammonium chloride (CTAC) solution (0.08 M). Then, the reducing agent composed of NaOH (0.28 mL, 0.1 M) and ascorbic acid solution (0.28 mL, 0.1 M) was injected *via* vigorously stirring. Here, the concentrations of HAuCl_4_ and H_2_PdCl_4_ were determined to ensure the proportion of Au and Pd in the alloy shell. The Au_*x*_Pd_1−*x*_ shell was controlled by adjusting *Y* and *Z* in the range of 0–2.5 mM (Table S1, ESI†).

Taking the fabrication of AuBP@Au_0.8_Pd_0.2_ nanostructures for instance, AuBP@Au_0.8_Pd_0.2_ nanostructures were synthesized by simultaneously adding the precursors (HAuCl_4_ (1 mM) and H_2_PdCl_4_ (0.25 mM)) into an AuBPs seed colloid (2.5 mL) containing CTAC, NaOH and ascorbic acid.

### Catalytic measurements

Firstly, it is essential to prepare 4-NP solution (0.125 mM) and fresh NaBH_4_ solution (0.1 M). Originally, 2 mL 4-NP and 1 mL ice-cold NaBH_4_ were added into cuvettes which resulted in the change of the solution from color-less to bright yellow. The solution was vigorously stirred for 5 min. Then, the AuBP@Au_*x*_Pd_1−*x*_ colloid was added and the mixture was stirred for a few seconds. During the catalytic reaction using different AuBP@Au_*x*_Pd_1−*x*_ colloids, an equivalent concentration of the nanocatalyst is ensured. The absorption spectra were recorded to monitor the reaction process in the spectral range of 275–500 nm.

### Electrochemical measurements

Electrochemical performances were studied on a CHI760D potentiostat using a typical three-electrode configuration. A saturated Ag/AgCl electrode, Pt foil, and glassy carbon (GC) electrode coated with prepared catalysts were applied respectively as the reference electrode, counter electrode, and working electrode. Before the electrochemical test, two steps are essential. Firstly, the glassy carbon electrode was polished with Al_2_O_3_, which is made of a mixture of deionized water (0.5 mL) and α-Al_2_O_3_ (0.5 μm) microspheres. After this step, the catalyst was dispersed into Nafion (0.5 wt%, 2.5 μL), DMF-C (2.5 μL) and deionized water (22.5 μL) by ultrasonication for 30 min to form an ink, and the catalyst ink (4 μL) was then pipetted onto the GC surface and dried at room temperature.

Before each electrocatalytic measurement, N_2_ was purged into the system for 30 min to remove the dissolved oxygen and achieve an N_2_-saturated atmosphere. The sweep rate was fixed at 50 mV s^−1^. Moreover, the activity was measured in an N_2_-saturated aqueous 0.3 M KOH solution. All electrochemical tests were conducted at room temperature.

### Characterization

Ultraviolet-visible-near-infrared (UV-vis-NIR) absorption spectra were recorded using a UV-3600 Plus ultraviolet/visible/near-infrared spectrophotometer with plastic cuvettes of 0.5 cm optical path length. Microscopic images were taken using a transmission electron microscope (TEM, JEOL-100CX, Japan). High-angle annular dark-field scanning transmission electron microscopy (HAADF-STEM) characterization and elemental mapping were performed on an FEI Tecnai F20 microscope operating at 200 kV and equipped with an Oxford energy-dispersive X-ray (EDX) analysis system. Inductively coupled plasma atomic emission spectrometry (ICP-AES) measurements were performed on an Agilent ICP-MS 7500a system. Our electrochemical performances were evaluated using a CHI760D workstation (CH Instruments, China).

## Results and discussion

**Fig. 1:**
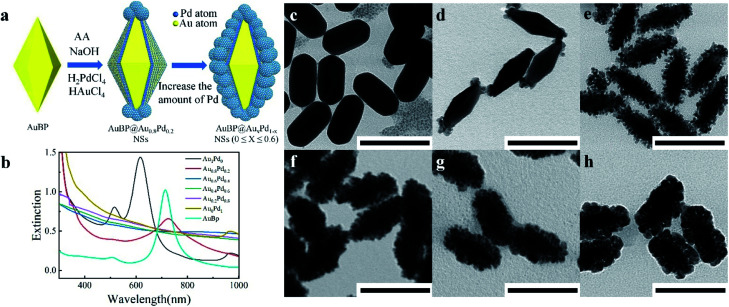
(a) Schematic illustration of the synthetic process of AuBPs@Au_*x*_Pd_1−*x*_ nanostructures. (b) UV-vis-NIR absorption spectra of AuBPs@Au_*x*_Pd_1−*x*_ nanostructures with increasing Pd in the alloyed shell. (c–h) The corresponding TEM images of the AuBPs@Au_*x*_Pd_1−*x*_ nanostructures obtained at 65 °C with the addition of 0.4 mL HAuCl_4_ and 0.4 mL H_2_PdCl_4_ (scale bars: 100 nm).

Due to their excellent chemical inertness, Au based nanostructures have prominent electrochemical stability. Meanwhile, the unique structural features enable AuBPs with fascinating SPR optical properties (Fig. S1a and b, ESI†), as reported previously.^30,31^ Consequently, AuBPs are chosen as a substrate to construct AuBPs@Au_*x*_Pd_1−*x*_ through improving the synthesis process. Fig. 1a illustrates the typical synthetic procedure, in which 0.4 mL HAuCl_4_ (*Y* mM) and 0.4 mL H_2_PdCl_4_ (*Z* mM) of different molar concentrations were added slowly and simultaneously into the AuBP colloidal solution containing CTAC, NaOH, and ascorbic acid at 65 °C. Fig. 1b shows the extinction spectra of the AuBP sample and AuBPs@Au_*x*_Pd_1−*x*_ nanostructures produced after adding different volume concentrations of HAuCl_4_ and H_2_PdCl_4_. The absorption peak at 720 nm is attributed to the characteristic SPR of AuBPs. Meanwhile, this SPR peak of the AuBPs can still be detected with a slight red shift in the extinction spectra of the AuBPs@Au_*x*_Pd_1−*x*_ nanostructures (at a certain Au/Pd ratio). Then the characteristic SPR of AuBPs dampened and disappeared for AuBP@Au_0.6_Pd_0.4_ owing to the increase of the Pd nanocluster shell on the surface of AuBPs. Compared with the as-prepared AuBPs, the overgrowth of Au on the surface of AuBPs into stumpy nanorods, *i.e.* AuBPs@Au_1_Pd_0_, leads to the SPR blue shift.

Fig. 1c–h show the representative images of AuBPs@Au_*x*_Pd_1−*x*_ nanostructures at 65 °C with the addition of 0.4 mL of HAuCl_4_/H_2_PdCl_4_ each. Deposition of alloyed Au/Pd nanoclusters on the AuBP surface greatly increases the surface area of the AuBPs. Fig. S2a, ESI† shows the optical spectra and corresponding TEM images of AuBPs@Au_*x*_Pd_1−*x*_ nanostructures obtained with decreasing Au/Pd contents. Gerald *et al.*^32^ also identified isolated agglomerates on the support with increasing Au atomic percentage. For our cases, when the numerical density of Pd atoms increases up to the critical point, they have a strong tendency to grow into Pd nuclears attached on the surface of AuBPs. As shown in Fig. S2b–e, ESI,† the Pd shell is constituted by numerous tiny dendrite-like nanocrystals. TEM images demonstrate that with increasing Au and Pd precursor content the Au and Pd are deposited firstly on the tips of AuBPs, and then attached on the side surface of AuBPs.

**Fig. 2:**
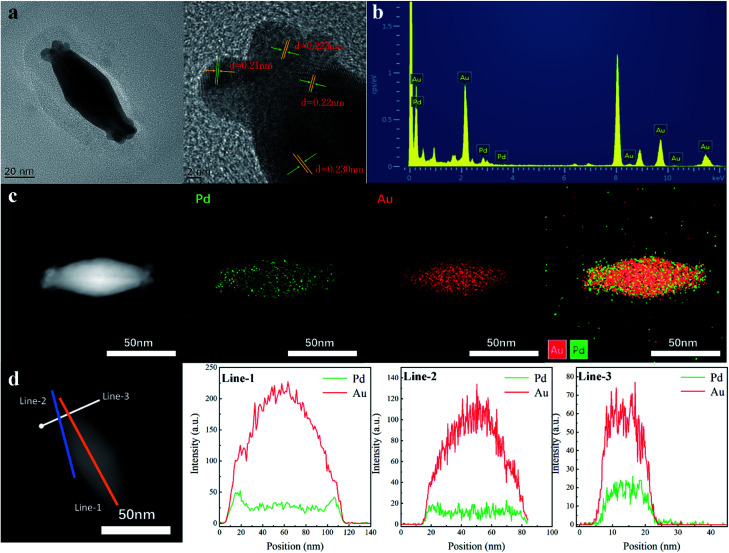
(a) Typical HRTEM images of AuBPs@Au_0.8_Pd_0.2_ nanostructures. (b) EDX spectrum of the AuBPs@Au_0.8_Pd_0.2_ nanostructures. (c) HAADF-STEM image and EDX elemental mapping patterns of the AuBPs@Au_0.8_Pd_0.2_ nanostructures. (d) EDX line scanning profiles of the AuBPs@Au_0.8_Pd_0.2_ nanostructures.

The AuBP core and Au/Pd alloyed shell were further investigated by high-resolution transmission electron microscopy (HRTEM). The HRTEM image shows a continuous lattice fringe pattern with a fringe interval of *ca.* 0.223 nm, which is indexed to the (111) facet of the face centered cubic Pd crystal (0.224 nm). Measurements showed that the obvious Au (111) plane with 0.235 nm fringe space was observed at the core position. As shown in Fig. 2b, obvious Au and Pd signals are observed in the EDX spectrum, confirming the deposition of Au and Pd atoms on the surface of AuBPs. The result of ICP-AES is well matched with the Au/Pd atomic ratio of AuBPs@Au_*x*_Pd_1−*x*_ nanostructures (note: the mole fraction of Pd in Table S2† (column-1) is the expression we use in our work but not the Pd actual amount) (Table S2, ESI†).

To further confirm the deposition sites of Au and Pd atoms, HAADF-STEM imaging and elemental mapping were performed to examine the detailed structure and elemental distributions of the AuBPs@Au_*x*_Pd_1−*x*_ nanostructures (Fig. 2c). From the EDS mapping image, the Pd and Au elements are well distributed in the selected area of AuBPs@Au_*x*_Pd_1−*x*_ nanocatalysts. These AuBPs were enveloped with Au/Pd shells into AuBPs@Au_*x*_Pd_1−*x*_ nanostructures, while the Au atoms at the center maintain the bipyramid shape. The center of the particle is pure Au, whereas Pd is concentrated at the outer edge of the nanostructures, which is supported by analysis across the nanostructures or the surface of the nanostructures. Signals of Pd (green line) and Au (red line) can be observed clearly in the EDX line-scan image. This observation suggested that Pd atoms prefer to deposit at the ends rather than on the side surface of AuBPs at low Pd/Au ratios, which can also support the slight red shift of SPR of AuBPs in Fig. 2a.

**Fig. 3:**
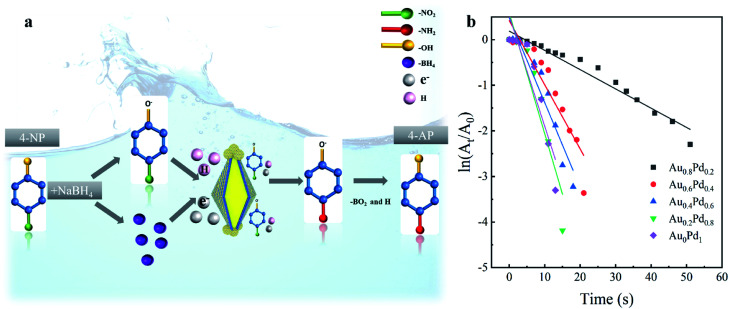
(a) Schematic illustration of the catalysis mechanism for AuBPs@Au_*x*_Pd_1−*x*_ nanocatalysts. (b) ln(*A*_*t*_/*A*_0_) *vs.* time plot for the determination of the rate constants of core–shell nanocatalysts with AuBPs@Au_0.8_Pd_0.2_, AuBPs@Au_0.6_Pd_0.4_, AuBPs@Au_0.4_Pd_0.6_, AuBPs@Au_0.2_Pd_0.8_, AuBPs@Au_0_Pd_1_.

The catalytic activities of different AuBPs@Au_*x*_Pd_1−*x*_ nanocatalysts were evaluated by the reduction of 4-NP to 4-AP with the presence of NaBH_4_. The reaction of 4-NP to 4-AP does not occur without catalysts but proceeds rapidly in the presence of metallic surfaces.^33^Fig. 3a illustrates the synergetic mechanism of AuBPs@Au_*x*_Pd_1−*x*_ in the catalytic reaction. With the presence of AuBPs@Au_*x*_Pd_1−*x*_ nanocatalysts in the system, the reaction of 4-NP to 4-AP can be confirmed by the increase of the absorption peak at ∼300 nm and the peak decrease of 400 nm, as shown in Fig. S3, ESI.† Because the concentration of NaBH_4_ added in the system is excessive in comparison with that of 4-NP, it can be assumed that the concentration of NaBH_4_ remain unchanged during the reaction. For different AuBPs@Au_*x*_Pd_1−*x*_ nanocatalysts, a linear relationship is followed between the logarithm of relative absorption intensity (ln(*A*_*t*_/*A*_0_), where *A*_*t*_ is the nanocatalyst actual reaction times) (Fig. 3b). Briefly, with decreasing Pd in the shells of five nanocatalysts (AuBPs@Au_0_Pd_1_, AuBPs@Au_0.2_Pd_0.8_, AuBPs@Au_0.4_Pd_0.6_, AuBPs@Au_0.6_Pd_0.4_, and AuBPs@Au_0.8_Pd_0.2_), the completion times of 4-NP reduction to 4-AP are respectively 13 min, 15 min, 18 min, 21 min, and 51 min, as illustrated by Fig. S3, ESI.†

In addition to the effect of Pd content on the catalytic reaction, it is also known that the catalytic activity was progressively improved by the increased specific surface area; on the surface there are abundant single-atomic steps and kinks with high catalytic activity, as reported previously.^34^ As shown in Fig. 1 and S2, ESI,† the surfaces of the nanocatalysts become more and more rough with the increase of Pd atom content. Thus, a large specific surface area plays an important role in improving the catalytic properties. With the advantages of the plasmonic effect, stable Au/Pd nanoclusters and a large specific surface area, AuBP@Au_*x*_Pd_1−*x*_ nanocatalysts are suitable for efficient catalytic applications.

**Fig. 4:**
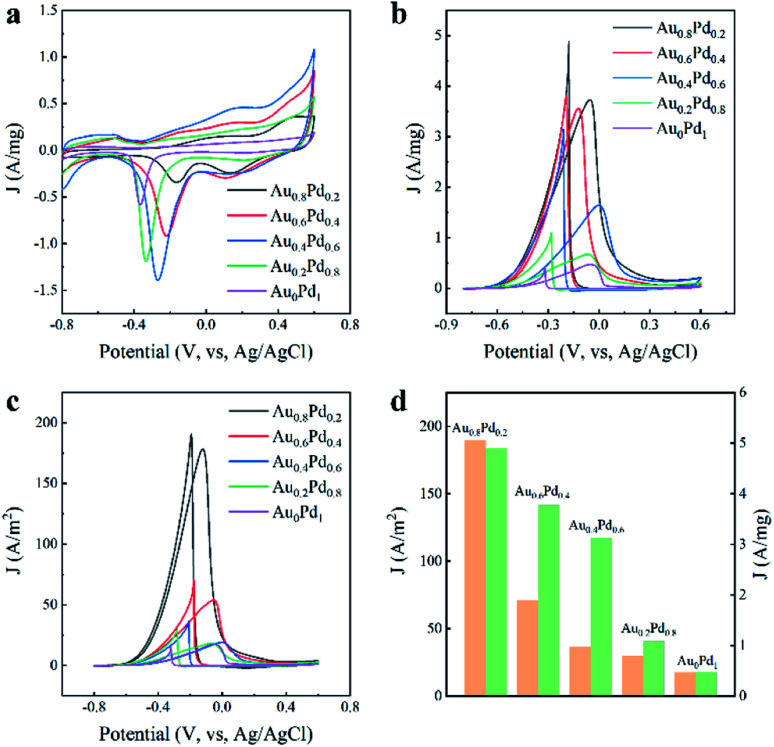
(a) CV curves for the AuBPs@Au_*x*_Pd_1−*x*_ nanocatalysts in N_2_-saturated 0.3 M KOH solution at a scan rate of 50 mV s^−1^. (b) CV curves for the AuBPs@Au_*x*_Pd_1−*x*_ nanocatalysts in N_2_-saturated KOH (0.3 M) solution containing ethanol (0.5 M) at a scan rate of 50 mV s^−1^. (c) CV curves of the ECSAs. (d) Specific activities (orange) and mass activities (green) of AuBPs@Au_*x*_Pd_1−*x*_ nanocatalysts. Note: AuBPs@Au_*x*_Pd_1-*x*_ nanocatalysts were obtained with the addition of 0.4 mL HAuCl_4_ and 0.4 mL H_2_PdCl_4_.

Pd-based nanocatalysts are appealing electrocatalysts toward the oxidation of alcohol indirect fuel cells.^35–37^ The electrochemical characteristics of the AuBPs@Au_*x*_Pd_1−*x*_ nanocatalysts were examined to identify reactions that occur on the electrode surface. Cyclic voltammetry (CV) curves were first measured in N_2_-saturated KOH (0.3 M) at a scan rate of 50 mV s^−1^, as displayed in Fig. 4a. The CV of the catalysts consists of two parts: the forward scan and the reverse scan. There were three peaks for potential during the positive sweep for the AuBPs@Au_*x*_Pd_1−*x*_ electrodes, which correspond to three different electrochemical processes. From left to right, the first peak between −0.8 V and −0.65 V is associated with hydrogen adsorption/desorption processes on the electrodes.^38^ The second peak between 0 v and 0.2 v indicates the oxidation of Pd atoms to PdO in electrooxidation. The third peak is the end of the oxidation line, which is attributed to the formation of gold oxides or adsorption of OH^−^ species on the Au nanostructure surface. And the existence of Au changes the redox properties of formation/reduction of PdO.^39^ The reduction curves between −0.4 v and 0.0 v correspond to the reduction of PdO during electrocatalytic measurements.^40,41^

The electrocatalytic performances of all AuBPs@Au_*x*_Pd_1−*x*_ nanocatalysts with different Au contents are summarized in Table 1 with the following characteristics: the onset mass of Pd and the electrochemical surface area. The electrochemically active areas (ECSA) of unit Pd are calculated using the equation (ECSA = *Q*/*m*_Pd_*C*) according to the desorption peak area of the oxygenated adsorption, in which *Q* and *C* refer to the total adsorbed oxygen charge and the charge density of mono layer adsorption of O atoms, respectively.^42^ The ECSA was calculated to be 1.99, 6.93, 8.61, 3.67, 2.70 m^2^ g^−1^ by quantifying the electric charges required for PdO reduction Table 1. All of the AuBPs@Au_*x*_Pd_1−*x*_ (0 < *x*< 1) samples exhibit an enlarged ECSA compared with AuBPs@Au_0.8_Pd_0.2_ (8.46 μg), and *m*_Pd_ increases along with the increase of Au/Pd atom ratios.

**Table 1:** The m_Pd_ and ECSA of the AuBPs@Au_*x*_Pd_1−*x*_ nanocatalysts

Catalyst	*m*_Pd_ μg	ECSA m^2^ g^−1^
Au_0.8_Pd_0.2_	8.46	1.99
Au_0.6_Pd_0.4_	13.25	6.93
Au_0.4_Pd_0.6_	25.15	8.61
Au_0.2_Pd_0.8_	50.88	3.67
Au_0_Pd_1_	89.81	2.70

All of the AuBPs@Au_*x*_Pd_1−*x*_ electrodes show two well-defined current characteristic peaks in the electrooxidation of ethanol, as shown in Fig. 4b and c. Each CV curve of nanocatalyst with area activity/mass activity is divided into two curves: forward scan and backward scan. The former results from ethanol oxidation during the forward scan, and the latter results from the oxidation of carbon-containing intermediates that are derived from the incompletely oxidized ethanol during the backward scan.^42^

CV measurements were performed in a N_2_-saturated mixture of KOH (0.3 M) and ethanol (0.5 M). The hydrogen desorption/adsorption region is suppressed in the presence of voltage between −0.8 V to −0.4 V due to the addition of ethanol to the solution. The anodized peak current with the AuBPs@Au_0.8_Pd_0.2_ (4.90 A mg^−1^ and 189.72 A m^−2^) catalyst is almost ten times that of the AuBPs@Au_0_Pd_1_ (0.47 A mg^−1^ and 17.48 A m^−2^). The significantly high anodic peak current for the ethanol electro-oxidation reaction indicated that the AuBPs@Au_0.8_Pd_0.2_ nanostructure is superior to the other AuBPs@Au_*x*_Pd_1−*x*_ nanocatalysts for ethanol oxidation.

For comparison, the specific activities and mass activities of different AuBPs@Au_*x*_Pd_1−*x*_ nanocatalysts are summarized in Fig. 4d. The mass activities of the five nanocatalysts were calculated to be 4.90, 3.78, 3.13, 1.08, 0.47 A mg^−1^, as shown in Fig. 4d. It showed that the mass activities of AuBPs@Au_0.8_Pd_0.2_, AuBPs@Au_0.6_Pd_0.4_, AuBPs@Au_0.4_Pd_0.6_, and AuBPs@Au_0.2_Pd_0.8_ are respectively 10.42, 8.04, 6.65, and 2.29 times that of AuBPs@Au_0_Pd_1_ respectively. Fig. 4d demonstrates that the specific activity of the AuBPs@Au_0.8_Pd_0.2_ nanocatalyst (189.72 A m^−2^) was 10.85 times greater than that of the AuBPs@Au_0_Pd_1_ nanocatalyst (17.48 A m^−2^). Thus, the AuBPs@Au_0.8_Pd_0.2_ nanocatalyst showed significant electrocatalytic performance compared to the other AuBPs@Au_*x*_Pd_1−*x*_ nanocatalysts. There is a volcano between the mass/specific activities and the Au/Pd atom ratios which indicates that AuBPs@Au_0.8_Pd_0.2_ exhibits optimized mass/specific activities. For AuBPs@Au_0.8_Pd_0.2_ with Pd nanoclusters depositing at two ends of AuBPs and exposing the AuBP surface, this structure is in favour of transferring the OH^−^ adsorbed on the Pd sites to the AuBP surface quickly. Meanwhile, the addition of Pd and Au reduces the charge transfer resistance and promotes the electrocatalytic activity towards the ethanol molecule. The electrochemical results showed that the catalytic activity depends on the particle size, shape and support. For other AuBPs@Au_*x*_Pd_1−*x*_ nanocatalysts, the mass/specific activities are presented in Fig. S4a–c, ESI.† When the amounts of HAuCl_4_ and H_2_PdCl_4_ were increased from 0.2 mL to 0.4 mL, the mass/specific activities of AuBPs@Au_*x*_Pd_1−*x*_ nanocatalysts were improved (Fig. S5a–c, ESI†).

Interestingly, with the increase of the Pd content in the AuBPs@Au_*x*_Pd_1−*x*_ nanostructures, the electrocatalytic activity for the catalytic oxidation decreases. Some possible explanations are proposed for the better performance of the AuBPs@Au_0.8_Pd_0.2_ nanocatalyst: (1) the increase of Pd oxides inhibits the adsorption of reactants. (2) The multiple twinned boundaries with more defects on the surface could supply more sites for the electro-oxidation, which is observed in the lower Pd content sample, *i.e*., AuBPs@Au_0.8_Pd_0.2_. (3) More active sites formed in the surface, such as edges, corners, and potential high-index facets on the surface crystals. The result is in agreement with the literature and confirms the promoting effects.^43^ This interesting result means that AuBPs@Au_*x*_Pd_1−*x*_ nanostructures with a low Pd account exhibit great potential as less expensive non-Pt electrocatalysts for ethanol electrooxidation in alkaline media.^44^

**Fig. 5:**
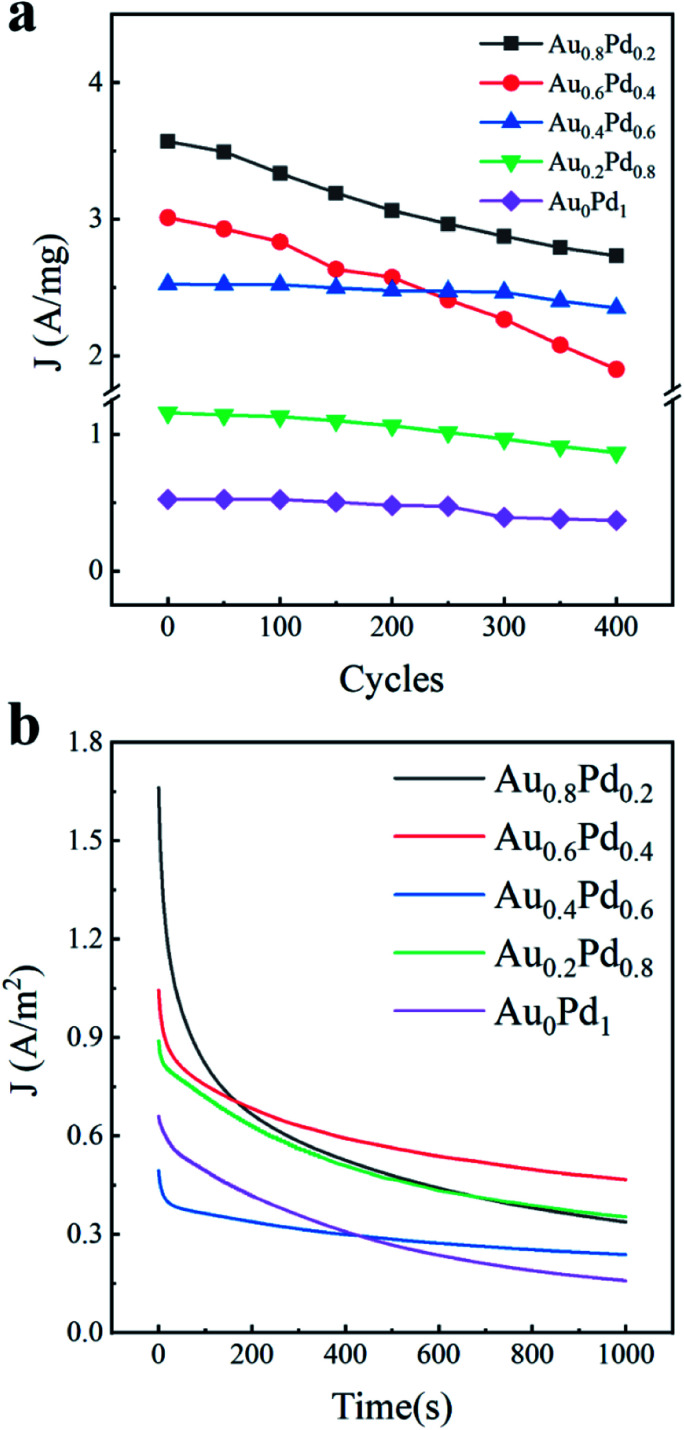
(a) Cycling measurement for different samples. (b) Chronoamperometric curves in 0.5 M ethanol + 0.3 M KOH solution for 1000 s at −0.23 V *versus* Ag/AgCl potential.

In addition to the activity, the durability of electrocatalysts is also an important factor to assess the catalytic performance. We therefore examined the stability of AuBPs@Au_*x*_Pd_1−*x*_ nanostructures by both the accelerated CV curves and chronoamperometric (CA) technique. At a fixed scan rate of 50 mV s^−1^ for 400 cycles between −0.8 V and 0.6 V, the cycling durability of the electrocatalysts was evaluated by repeatedly running CV tests (Fig. 5a). The 400 lap cycle curves of AuBPs@Au_0.8_Pd_0.2_ nanocatalysts and AuBPs@Au_0.6_Pd_0.4_ nanocatalysts decreased rapidly and the other nanocatalyst curves fell very slowly, but the AuBPs@Au_0.8_Pd_0.2_ 400 lap cycle curve was still slightly higher than that of other nanocatalysts. It is very interesting that the loss in the current density of the AuBPs@Au_0.8_Pd_0.2_ nanocatalyst was as high as that of the other nanocatalysts even after 400 cycles despite their low density of under coordinated surface atoms. Compared with the other electrodes, AuBPs@Au_0.8_Pd_0.2_ exhibits much higher initial polarization mass-specific current densities for ethanol electrooxidation.

In addition, the electrocatalytic stabilities of the five AuBPs@Au_*x*_Pd_1−*x*_ nanocatalysts were also investigated by CA measurements at a potential of −0.23 V for 1000 s (Fig. 5b). The five curves show a rapid decay in the current density at the initial stage of reactions and then a slow decay. The initial currents of AuBPs@Au_0.8_Pd_0.2_ are much higher than those of the other catalysts during the ethanol oxidation. It is well known that the current density decay is possibly caused by catalyst poisoning by CO^−^ species formed in alcohol oxidation and the PdO formation.^45,46^ At the end of the reactions, current densities of 1.9, 3.59, 1.60, 1.71 and 1.55 A m^−2^ are obtained by AuBPs@Au_0.8_Pd_0.2_, AuBPs@Au_0.6_Pd_0.4_, AuBPs@Au_0.4_Pd_0.6_, AuBPs@Au_0.2_Pd_0.8_, and AuBPs@Au_0_Pd_1_, respectively. The stability of the electrocatalysts with 0.2 mL HAuCl_4_ and 0.2 mL H_2_PdCl_4_ precursors was evaluated by running repeated CV tests at a fixed scan rate (50 mV s^−1^) (Fig. S6a–c, ESI†).

Interestingly, the oxidation current on the AuBPs@Au_0.6_Pd_0.4_ nanocatalysts is higher than those on the other nanostructures at the end of measurement, and the current decay is the slowest among the various nanostructures. It is shown that AuBPs@Au_0.6_Pd_0.4_ nanocatalysts maintained the most stable electrocatalytic performance. Taken together, these results indicate that AuBPs@Au_0.8_Pd_0.2_ shows a better electrocatalytic activity than others, but its electrocatalytic stability is not as good as the other nanocatalysts. These differences indicated the durability of AuBPs@Au_*x*_Pd_1−*x*_ nanocatalysts with a low Pd content is very sensitive to the electrochemical measurement mode in electrocatalysis, which is a significant aspect to be studied in the future. Further investigations on enhancing the electrocatalytic stability toward ethanol oxidation using low Pd nanocatalysts are currently underway.

## Conclusions

In summary, we have successfully prepared AuBPs@Au_*x*_Pd_1−*x*_ nanocatalysts by adjusting the precursor concentrations of HAuCl_4_ and H_2_PdCl_4_. The formation of an Au/Pd core–shell structure was proved by HAADF-STEM imaging and elemental mapping. The EDX spectrum confirmed the presence of metallic Pd and Au. Electrochemical measurements indicated that the activity of catalytic oxidation of ethanol depends on the catalyst structure. Precisely designing the amount of HAuCl_4_ and H_2_PdCl_4_ regulated the structure of Au/Pd, and optimized the oxygen adsorption of Au or Au/Pd. The AuBPs@Au_0.8_Pd_0.2_ nanostructure exhibited the highest catalytic activity in alkaline media compared with the other nanostructures. The specific activities and mass activities of AuBPs@Au_0.8_Pd_0.2_ are 10.85 times that of the AuBPs@Au_0_Pd_1_ nanocatalysts. Chronoamperometric tests revealed that the addition of the HAuCl_4_ precursor remarkably enhances the stability and electrocatalytic activity of AuBPs@Au_*x*_Pd_1−*x*_ due to the bifunctional effect of Au/Pd alloys. Herein, this work provides an excellent demonstration for the reasonable design of high efficiency electrooxidation nanocatalysts. The nanostructure has important research significance and promising application value in the field of catalysis, which will promote the development of ethanol fuel cells.

## Author contributions

Baihe Hanqi: formal analysis, investigation, project administration, visualization, writing – original draft, and writing – review & editing. Juan Xu: conceptualization, methodology, and validation. Xingzhong Zhu: data curation, funding acquisition, and resources. Caixia Kan: funding acquisition, resources, supervision, and writing – review & editing.

## Conflicts of interest

There are no conflicts to declare.

## Supplementary Material

NA-004-D1NA00878A-s001
