# Supplementary material for: Gold nanobipyramids doped with Au/Pd alloyed nanoclusters for high efficiency ethanol electrooxidation
Source: Nanoscale Adv. 2022 Mar 3;4(7):1827–34. doi: 10.1039/d1na00878a (PMC9417086; doi:10.1039/d1na00878a)
Supplement: NA-004-D1NA00878A-s001 [file NA-004-D1NA00878A-s001.pdf]

Electronic Supplementary Material (ESI) for Nanoscale Advances.
This journal is © The Royal Society of Chemistry 2022

Electronic Supplementary Information

Gold nanobipyramid dopped with Au/Pd alloyed nanoclusters for high efficiency ethanol electrooxidation

Baihe Hanqi,[a] Juan Xu,[a] Xingzhong Zhu[a,b] and Caixia Kan[a,b,*]

[a] *College of Science, Nanjing University of Aeronautics and Astronautics, Nanjing 210016, China. E-mail: cxkan@nuaa.edu.cn;*

[b] *MIIT Key Laboratory of Aerospace Information Materials and Physics, Nanjing University of Aeronautics and Astronautics, Nanjing 211106, China*

* Email: cxkan@nuaa.edu.cn

Supplementary figures

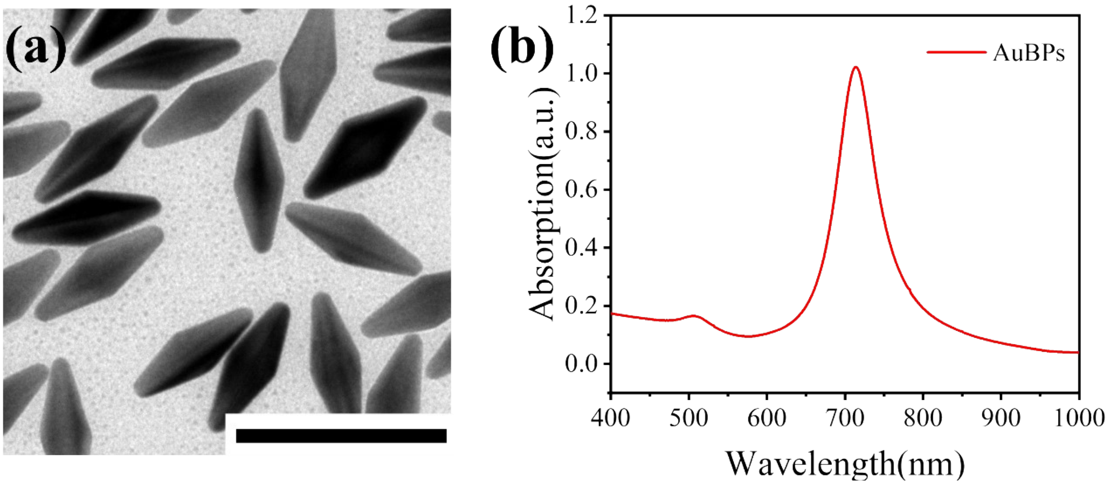

Fig. S1 (a) TEM image and (b) corresponding optical spectrum of the AuBPs (Scale bars: 100 nm)

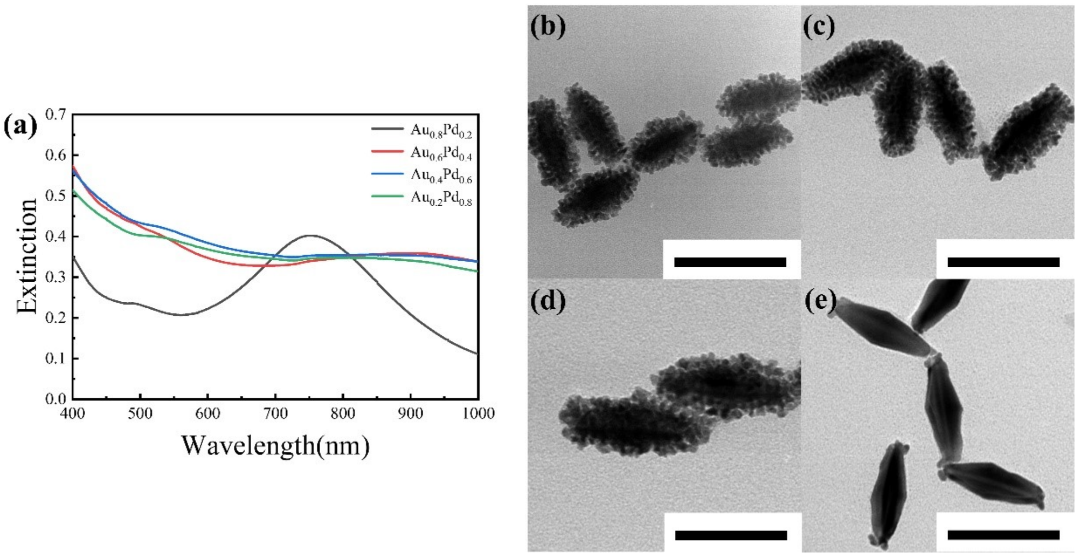

Fig. S2 (a) UV-vis-NIR absorption spectra of AuBPs@Au_xPd_{1-x} nanostructures with varying Au/Pd proportion in the alloy shell. TEM images of the nanostructures obtained under the addition of varying H_2PdCl_4 of 2mM (b), 1.5mM (c), 1mM (d) and 0.25mM (e) obtained at 65°C with respective addition of 0.2 mL $HAuCl_4$ and 0.2 mL H_2PdCl_4 (Scale bars: 100 nm).

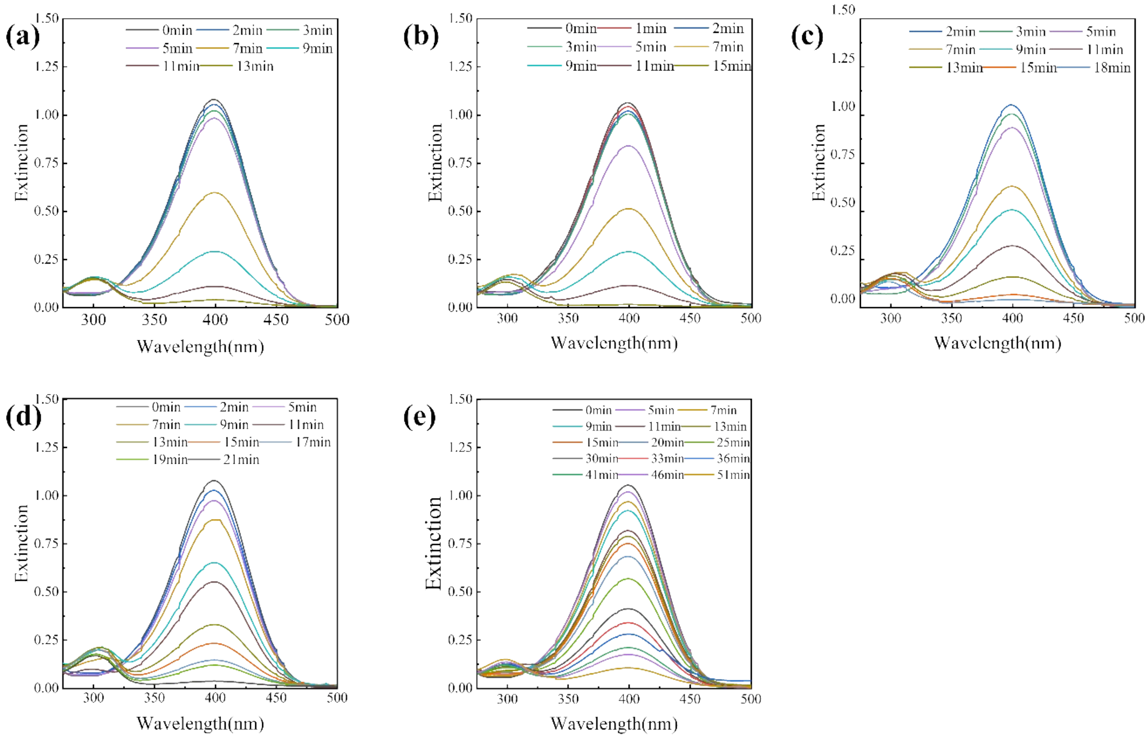

Fig. S3 Absorption spectra of 4-NP reduced by $NaBH_4$ in the presence of AuBPs@Au_xPd_{1-x}. (a) AuBPs@Au_0Pd_1, (b) AuBPs@$Au_{0.2}Pd_{0.8}$, (c) AuBPs@$Au_{0.4}Pd_{0.6}$, (d) AuBPs@$Au_{0.6}Pd_{0.4}$, (e) AuBPs@$Au_{0.8}Pd_{0.2}$.

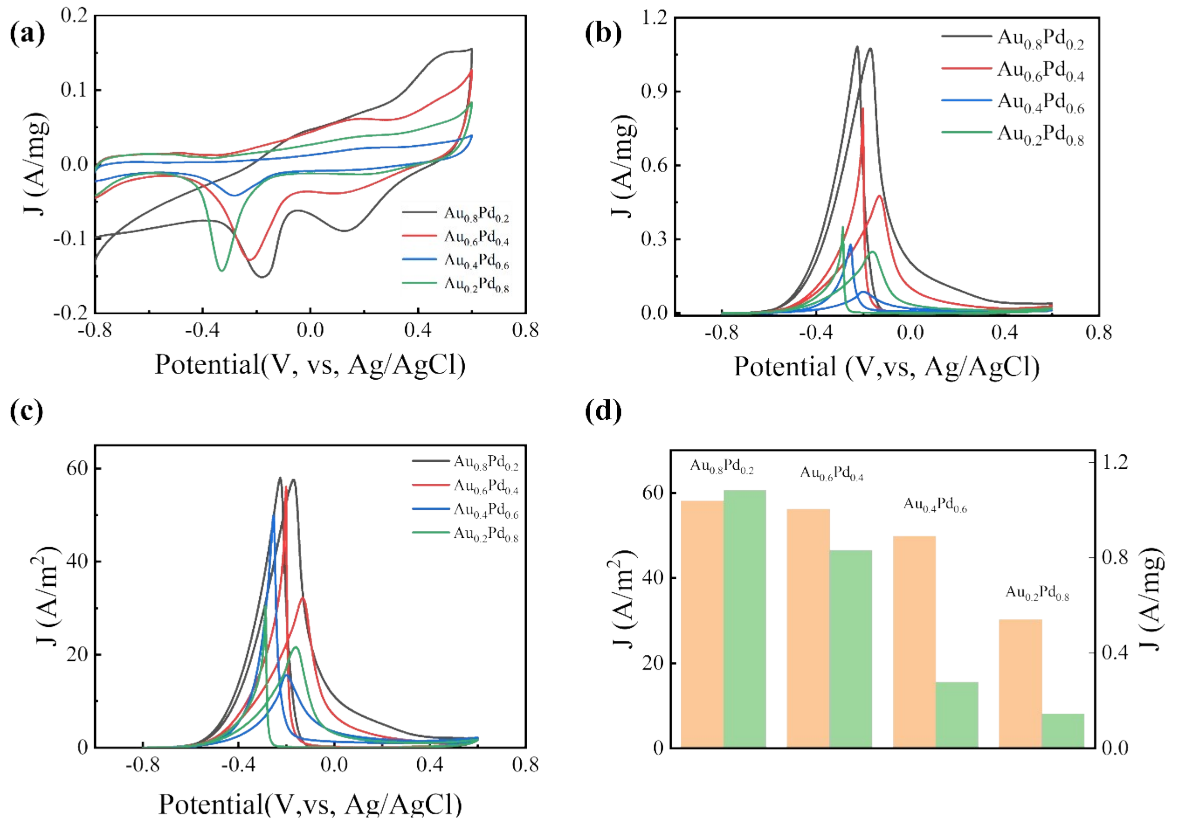

Fig. S4 (a) Cyclic voltammograms for the AuBPs@Au_xPd_{1-x} nanocatalysts in N_2-saturated KOH (0.3M) solution at a scan rate of 50 mV s^{-1}. (b) CV curves for the AuBPs@Au_xPd_{1-x} nanocatalysts in N_2-saturated KOH (0.3M) solution containing ethanol (0.5M) at a scan rate of 50 mV s^{-1}. (c) CV curves by the ECSAs. (d) Mass activities (green) and specific activities (orange) of AuBPs@Au_xPd_{1-x} nanocatalysts from measurement. Note: AuBPs@Au_xPd_{1-x} nanocatalysts were obtained with addition of 0.2 mL $HAuCl_4$ and 0.2 mL H_2PdCl_4.

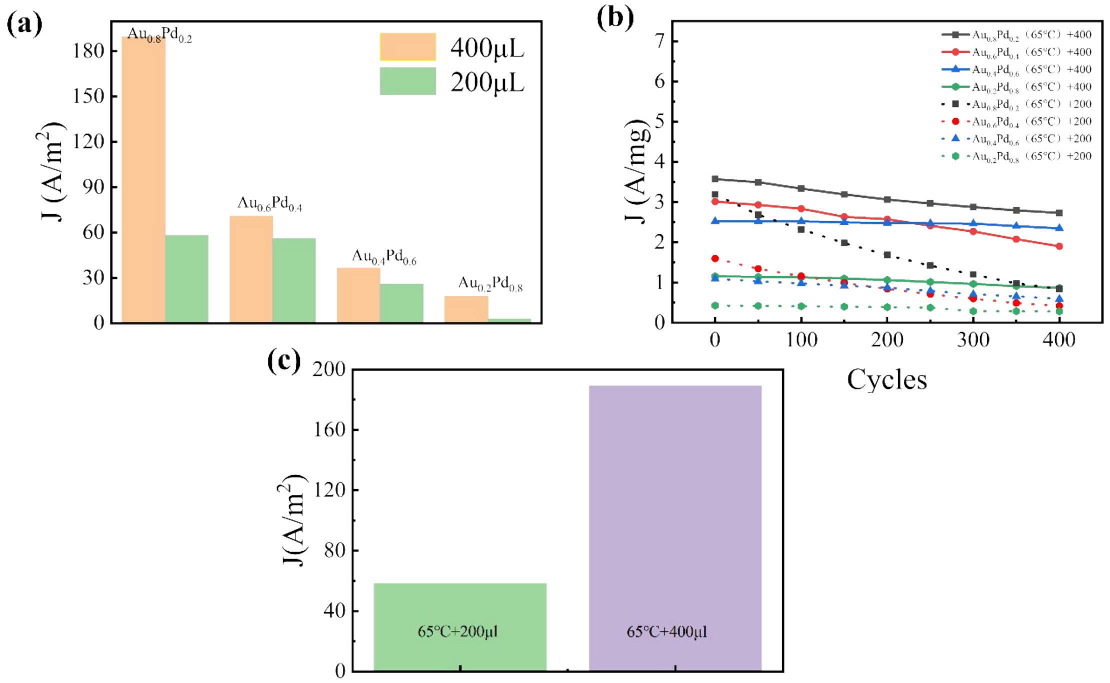

Fig. S5 (a) specific activity of the AuBPs@Au_xPd_{1-x} nanostructures obtained at 65°C with addition of $HAuCl_4$ /H_2PdCl_4 precursor (0.2 mL and 0.4 mL). (b) Cycling measurement of the AuBPs@Au_xPd_{1-x} nanocatalysts obtained at 65°C with addition of $HAuCl_4$ /H_2PdCl_4 precursor (0.2 mL and 0.4 mL). (c) specific activity of the AuBPs@$Au_{0.8}Pd_{0.2}$ nanocatalysts obtained at 65°C with addition of different volume of $HAuCl_4$ /H_2PdCl_4 precursor (0.2 mL and 0.4 mL).

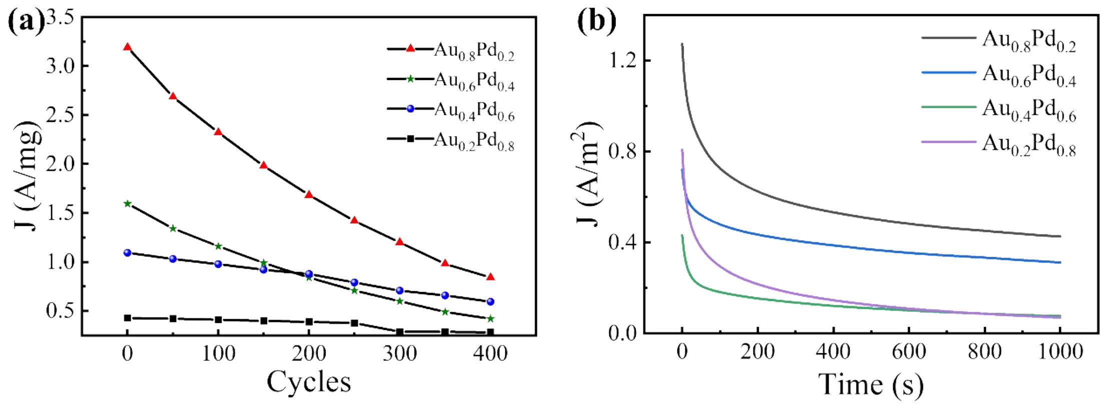

Fig. S6 (a) Cycling measurement for AuBPs@Au_xPd_{1-x} nanocatalysts with addition of 0.2 mL $HAuCl_4$ /H_2PdCl_4 precursor. (b) Chronoamperometric curves in mixture solution of KOH (0.3M) and ethanol (0.5M) for 1000s at -0.23V versus Ag/AgCl potential.

Table S1 The AuBPs@Au_xPd_{1-x} with different concentration for Au and Pd precursors

Sample	Concentration of H_2PdCl_4 (mM)	Concentration of $HAuCl_4$ (mM)	Amount of H_2PdCl_4 (mL)	Amount of $HAuCl_4$ (mL)
**AuBPs@$Au_{0.8}Pd_{0.2}$**	1	0.25	0.4	0.4
**AuBPs@$Au_{0.6}Pd_{0.4}$**	1	1.5	0.4	0.4
**AuBPs@$Au_{0.4}Pd_{0.6}$**	1.5	1	0.4	0.4
**AuBPs@$Au_{0.2}Pd_{0.8}$**	0.5	2	0.4	0.4
**AuBPs@Au_0Pd_1**	2.5	0	0.4	0.4

Table S2 Comparison of the AuBPs@Au_xPd_{1-x} with different mole fractions for Au and Pd precursors.

Sample	Pd amount of substance concentration (mg/ L)	Pd mass percent	Au/Pd atomic ratio
**AuBPs@$Au_{0.8}Pd_{0.2}$**	0.32	40.5%	44.2:55.8
**AuBPs@$Au_{0.6}Pd_{0.4}$**	0.50	50.0%	35.0:65.0
**AuBPs@$Au_{0.4}Pd_{0.6}$**	0.96	65.3%	25.5:74.5
**AuBPs@$Au_{0.2}Pd_{0.8}$**	1.92	80.3%	11.6:83.4
**AuBPs@Au_0Pd_1**	3.02	82.9%	9.9:90.1
